# Regulation and biological functions of the CX3CL1-CX3CR1 axis and its relevance in solid cancer: A mini-review

**DOI:** 10.7150/jca.47022

**Published:** 2021-01-01

**Authors:** Selma Rivas-Fuentes, Alfonso Salgado-Aguayo, Jenny Arratia-Quijada, Patricia Gorocica-Rosete

**Affiliations:** 1 Department of Research on Biochemistry, Instituto Nacional de Enfermedades Respiratorias Ismael Cosío Villegas, Mexico City, Mexico.; 2 Laboratory of Research on Rheumatic Diseases, Instituto Nacional de Enfermedades Respiratorias Ismael Cosío Villegas, Mexico City, Mexico.; 3 Department of Biomedical Sciences, Centro Universitario de Tonalá, Universidad de Guadalajara, Tonalá Jalisco, Mexico.

**Keywords:** CX3CL1, CX3CR1, cancer, regulation

## Abstract

CX3CL1 is a transmembrane protein from which a soluble form can be generated by proteolytic shedding. Membranal and soluble forms of CX3CL1 exhibit different functions, although both bind to the CX3CR1 chemokine receptor. The CX3CL1-CX3CR1 axis mediates the adhesion of leukocytes and is also involved in cell survival and recruitment of immune cell subpopulations. The function of CX3CL1 is finely tuned by cytokines and transcription factors regulating its expression and post-translational modifications. On homeostasis, the CX3CL1-CX3CR1 axis participates in the removal of damaged neurons and neurogenesis, and it is also involved on several pathological contexts. The CX3CL1-CX3CR1 axis induces several cellular responses relevant to cancer such as proliferation, migration, invasion and apoptosis resistance. In this review, we address biological aspects of this molecular axis with important therapeutic potential, emphasizing its role in cancer, one of the most prevalent chronic diseases which significantly affect the quality of life and life expectancy of patients.

## Introduction

Accumulated evidence from over 20 years indicates that chemokines have an important role in various aspects of cancer 1. The chemokine family and its receptors are a promiscuous system; that is, a chemokine can bind to several receptors, and a chemokine receptor can be activated by several chemokines. Promiscuity represents an important challenge to develop the therapeutic potential of these molecules or their antagonists. Unlike most chemokines, in physiological concentrations CX3CL1 binds to a single receptor, CX3CR1, a characteristic that gives it significant therapeutic potential. CX3CL1 is commonly expressed in the tumor microenvironment and is capable of inducing cell migration in both tumor cells and immune cells with anti-tumor activity. In addition, activation of the CX3CL1-CX3CR1 axis can drive several pro-tumor cell responses. This review summarizes the current knowledge about the CX3CL1-CX3CR1 axis, its biological functions and regulation, as well as the information available in the literature about its role in cancer and its possible therapeutic utility. Therapeutic options for people living with certain forms of cancer remain limited, thus the importance of characterizing in detail new molecules that could be relevant to the pathophysiology of this disease.

## Molecular description of CX3CL1 and its receptor

**Figure 1:**
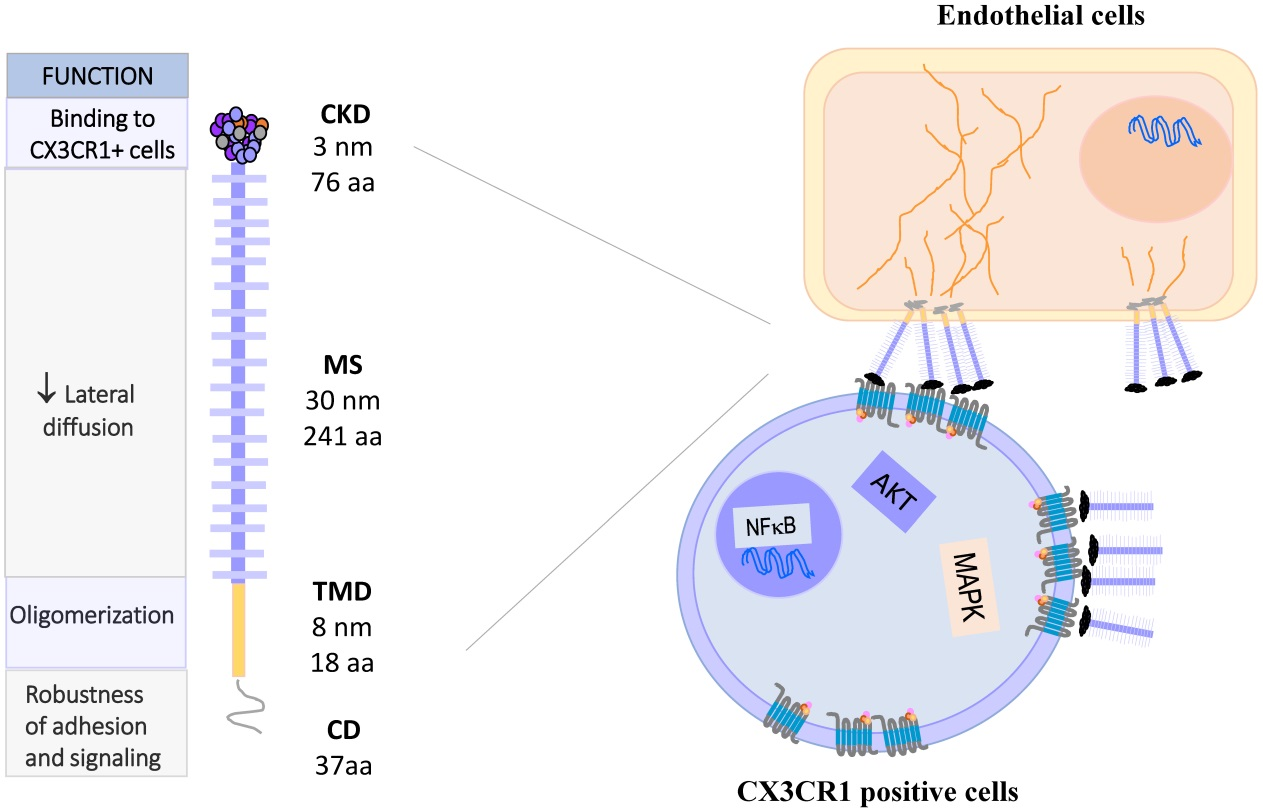
** CX3CL1: Molecule, oligomerization and signaling.** CX3CL1 is synthesized as a membrane bound molecule. It is composed of four domains: the chemokine binding domain (CDK), the mucin stem region (MS), the transmembrane domain (TMD) and the cytoplasmic domain (CD). Membrane-bound CX3CL1 forms constitutive oligomers, like CX3CR1. The binding of CX3CL1 with CX3CR1 induces the activation of heterotrimeric G proteins associated with this receptor and activates the signaling pathways of MAPK and AKT.

Fractalkine, or CX3CL1 according to the systematic chemokine nomenclature, is the only member of the CX3C chemokine family 2. It was originally named neurotactin by Bazan *et al*. because of its robust expression in the brain 3. CX3CL1 has a separation of three amino acids residues in its amino terminal cysteine ​​motif, instead of none or one in the CC and CXC families, respectively. Additionally, CX3CL1 has the peculiarity of being expressed as an anchored transmembrane protein, with the chemokine domain bound to the top of a mucin stalk **(Fig. 1 left)**. This structure endows CX3CL1 with peculiar biological characteristics, such as mediating cell adhesion 3. The native transmembrane CX3CL1 (mCX3CL1) can be cleaved by metalloproteinases (MMPs), producing a soluble CX3CL1 (sCX3CL1) that is chemotactic like other chemokines. This means that CX3CL1 has two molecular forms with different cellular functions: adherence and migration. Currently, only other chemokine is known to be synthesized as bound to the cell membrane by a mucin stalk: CXCL16, which also functions as cell adhesion molecule 4.

The gene coding for human CX3CL1 is located on chromosome 16q13 5, and has three exons. CX3CL1 is a type I membrane protein composed of 373 amino acids residues. It consists of 4 domains: a chemokine domain (CKD) with a length of 76 residues; a 241 residues long mucin stalk (MS, composed of 17 mucin repeats), a 19 residues long transmembrane domain (TM) and a cytosolic domain (CD) 37 residues long 6. Deterre *et al.* studied the molecular function of CX3CL1 domains. They generated CX3CL1 deletion mutants, which were expressed in both cell lines (HEK 293, COS7 and CHO) and primary cells (HUVEC). Their results indicate that the TM domain is responsible for the aggregation of several CX3CL1 molecules. On the other hand, a high glycosylation of the MS region limits the lateral movement of CX3CL1 in the cell membrane, causing a decrease in the diffusion of the molecule, which could favor its binding to CX3CR1 expressing cells. Finally, the cytoplasmic region strengthens the adhesion of CX3CL1 with its receptor, through its anchoring with cytoskeletal proteins 7. Biophysical studies (BRET, FRAP) indicate that mCX3CL1 forms aggregates in the cell membrane **(Fig. 1 right)** 7, 8.

sCX3CL1 is produced through proteolysis by MMPs such as ADAM17 (TACE) 9, 10, ADAM10 11 MMP-2 12 or cathepsin S 13. Once cleaved, an 85 KDa soluble fragment is usually generated 10. Crystallographic studies indicate that the chemokine domain can form dimers through folding of β sheets 14, influencing the way in which the chemokine domain associates with chemokine receptors.

CX3CL1 binds to an exclusive receptor called CX3CR1, which was identified in 1998 by Combadière* et al*. 15. However, an isolated report indicated that CX3CR1 binds CCL26, a chemokine involved on Th2 immune responses, leading to calcium mobilization and chemotaxis. The binding affinity of CCL26 to CX3CR1 is 10- 20 fold less compared to CX3CL1 16.

The gene coding for CX3CR1 is located on chromosome 3p22.2. It consists of four exons and three introns, and its expression is controlled by three promoters 17. Similar to other members of the chemokine receptor family, CX3CR1 is a 7-transmembrane receptor coupled to heterotrimeric G proteins (GPCRs). After ligand binding, the receptor associated-G protein is activated, and the α subunit dissociates from the βγ complex. This event activates several signaling pathways: PI3K kinases and MAPK kinases (JNK, ERK 1/2, p38), AKT (Ser-473 Thr-308), Src, and eNOS 18-24, which lead to different cellular responses such as migration, survival and apoptosis resistance **(Fig. 1, right)**.

**Figure 2:**
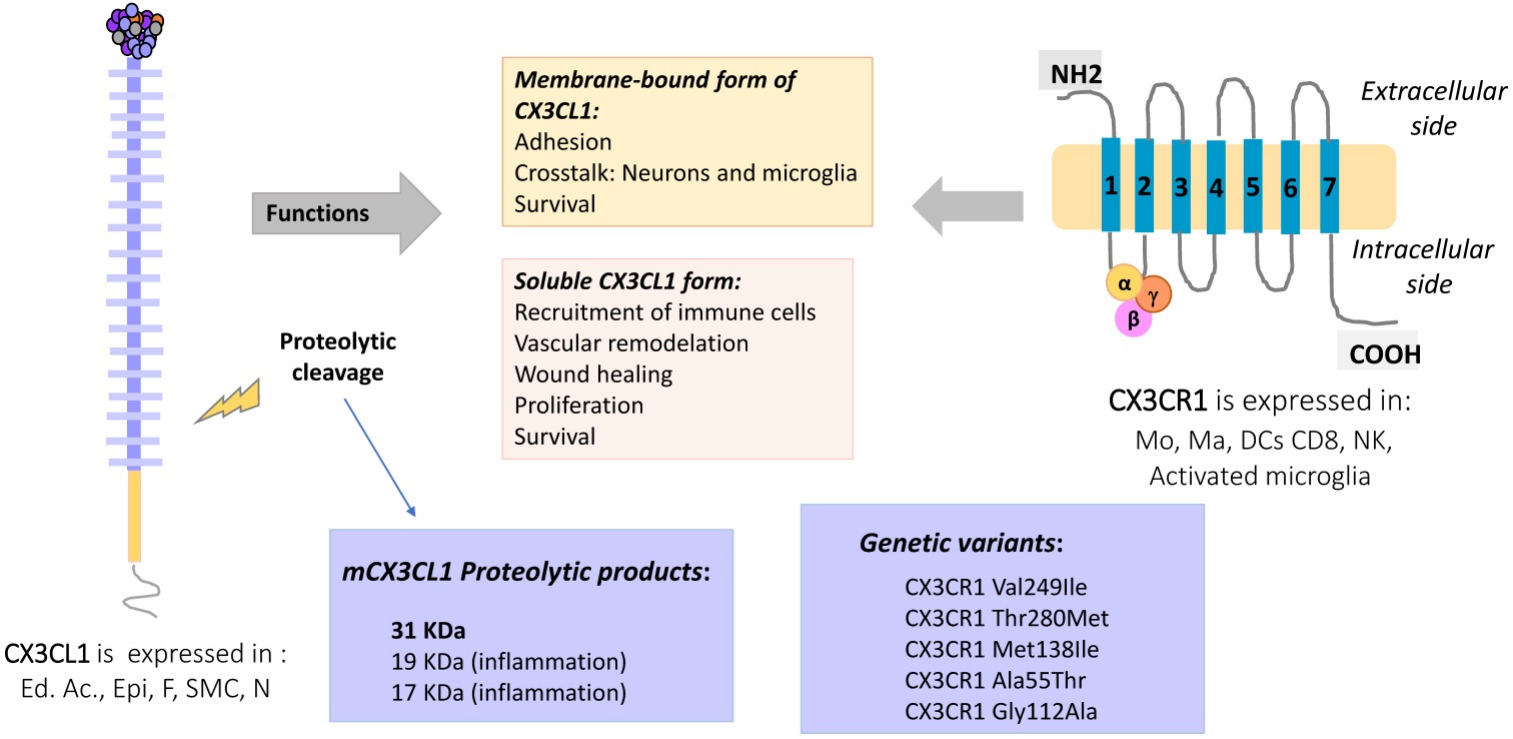
** CX3CL1-CX3CR1 Axis, molecular aspects and functions.** Membrane-bound CX3CL1 can be enzymatically shed, producing a soluble form of the molecule. At least three proteolytic products of CX3CL1 have been described, the most typical being that of 31 KDa. The membrane bound form is involved in adhesion and intercellular communication processes, while the CX3CL1 soluble form induces mainly proliferation, survival and other functional responses. The CX3CR1 receptor has 7 transmembrane domains and is coupled to heterotrimeric G proteins. Some polymorphic variants of CX3CR1 have been described as a risk factor for atherosclerosis. Abbreviations: Ed Ac, endothelium activated; Epi, Epithelial cells; F, fibroblast; SMC, smooth muscle cells; Neu, neurons, Mo, monocytes; Ma, macrophages; DC, dendritic cells; CD8, CD8 T cells; NK, Natural killer cells.

Several polymorphic variants of CX3CR1 have been described, such as V249-T280, I249-T280 and I249-M280, which present slight conformational changes 25 that partially explain their functional variations. For example, cells (human monocytes or CX3CR1 transfected cells) expressing different polymorphic variants of CX3CR1 were tested by the Parallel Plate Laminar Flow Assay and other adhesion techniques. The results showed that the I249-M280 CX3CR1 variant has modified adhesive properties with respect to the other variants 26, 27. This condition could be important in a pathophysiological context such as atherosclerosis. A clinical-epidemiological study reported that the I249-M280 CX3CR1 variant is a risk factor for the development of atherosclerotic plaques 28. **Figure 2** summarizes the molecular and functional characteristics of CX3CL1-CX3CR1.

## CX3CL1-CX3CR1: Expression and regulation

CX3CL1 is expressed in low levels in the liver and kidneys, with a robust expression in brain, colon, heart and lung 3. CX3CL1 is constitutively expressed by epithelial cells, dendritic cells (DCs), smooth muscle cells and neurons, among others; its expression is inducible on endothelial cells, fibroblasts, and astrocytes 29. The expression of CX3CL1 is regulated by cytokines such as IFN-γ, TNF-α, IL-1β and TGF-β, which alone or in specific combinations modify the expression of CX3CL1 or its receptor. For example, it has been reported that the combination of TNF-α and IFN-γ has a synergistic effect on the over-expression of CX3CL1 in HUVEC cells and osteoclasts 30, 31. This combination of cytokines also induces the expression of CX3CL1 in aortic smooth muscle cells, which under basal conditions do not express CX3CL1 32. In pulmonary fibroblasts the combination of IL-1β and IFN-γ has a synergistic effect on the expression of CX3CL1 33.

On the other hand, CX3CR1 is expressed by infiltrating immune cells such as monocytes, CD8^+^ T cells, and NK cells 3; and also, by tissue resident cells such as macrophages and DCs 34. CX3CR1 expression is positively regulated by TGF-β in rat microglia cells. Importantly, it was found that after induction of CX3CR1, CX3CL1 signaling was inhibited, highlighting the fact that a higher expression of CX3CR1 or its ligand after stimulation with a cytokine, is not always indicative of an increase in axis signaling 35. Hypoxia is another inductor of CX3CR1 expression in several prostate cancer cell lines 36.

The translation of CX3CL1 or CX3CR1 mRNA can be blocked by microRNAs. MicroRNAs are small RNA sequences of 19 to 22 nucleotides whose function is the silencing of target messenger RNA 37. It has been reported that miR-195 binds to the 3'-UTR region of CX3CL1; miR-195 has been related with the inhibition of proliferation and apoptosis on stromal endometrial cells 37. *In vitro* studies have shown that CX3CL1 is a direct downstream target of miR-561-5p, a microRNA that has been associated with pulmonary metastasis and a poor prognosis in hepatocellular carcinoma (HCC) patients. Relevantly, clinical samples exhibited a negative correlation between miR-561-5p expression and levels of CX3CL1 and CX3CR1^+^ in NK cells, cells that exhibit a relevant antitumor response 38, suggesting that CX3CL1 could have clinical relevance in cancer. Another microRNA that is known to regulate CX3CL1 is miR-29b, which has been linked to advanced stages of oral squamous cell carcinoma 39. Regarding CX3CR1, studies on several non-small cell lung cancer cell lines indicate that miR-296-3p reduces CX3CR1 levels 40; this microRNA is an attenuator of cell proliferation and its transfection increases the sensitivity of these cells to paclitaxel 40. Another study shows that miR-27a-5p inhibits the expression of CX3CR1 in human NK cells 41, although the clinical relevance of this result is unknown.

Once synthesized, CX3CL1 is potentially susceptible to post-translational modifications, a general phenomenon largely described for chemokines 42. Two posttranscriptional modifications have been described for CX3CL1: a modification by glutaminylcyclase that consists in modifying the glutamine of the first amino-terminal 43, and a proteolytic cleavage managed by specific enzymes like MMPs. The enzyme glutaminylcyclase produces pyroglutamate at the amino terminal (pGlu1-CX3CL1 modified with N-terminal). This modification stabilizes the binding of CX3CL1 to its receptor, which increases the signaling of ERK, AKT and p38 44. Interestingly, glutaminylcyclase isoenzymes are abundant in the brain (especially on a neuroinflammatory context), an organ that has a very robust expression of CX3CL1 45. *In vitro* studies have shown that under inflammatory conditions there is coexpression of CX3CL1 and glutaminylcyclase 44. Taken together, these results suggest that glutaminylcyclases may be important in regulating the functionality of the CX3CL1-CX3CR1 axis in inflammatory processes of the central nervous system. Another type of post-translational modification of CX3CL1 is the enzymatic release of a fragment of the chemokine domain; this modification can be regulated by specific cytokines that could vary according to the cell type, for example, in human fibroblasts the combination of IL-1β and IFN-γ increases the presence of soluble CX3CL1 in cultures 33. It has been reported that different enzymes can cut mCX3CL1 to produce its soluble form. For example, in COS-7 cells stimulated with PMA, the shedding is mediated by the enzyme ADAM-10 11; whereas in synovial fibroblasts, the proteolytic cleavage of CX3CL1 is mediated by the enzyme ADAM-17 (TACE), but not by ADAM-10 46, while in smooth muscle cells, the enzyme responsible for the shedding of CX3CL1 is cathepsin S 47. On the other hand, it has been recently reported that CX3CL1 is a promoter of the production of MMP-3 through the activation of CX3CR1 via c-Raf, MEK, ERK and NF-κB 48.

Experimental evidence has shown that CX3CL1 can induce its own expression: in rat aortic smooth muscle cells expressing both CX3CL1 and CX3CR1, CX3CL1 can upregulate the expression of both proteins via AKT (PI3K/PDK1/AKT/NIK/IKK/NF-kB). Autocrine induction of CX3CL1 increased proliferation and cellular adhesion, which could be relevant in the pathophysiology of atherosclerosis 49.

Another way through which chemokines function can be modified, is through their binding to glycosaminoglycans. Glycosaminoglycans (GAGs) are linear saccharide sequences, which when covalently linked to a protein core, form a proteoglycan. GAGs regulate the activity of different protein families such as cytokines, growth factors, enzymes, and chemokines, among other 50. They are abundant in the extracellular matrix and on the surface of cells. Chemokines bind mainly to GAGs of the heparan sulfate/heparin type. GAGs are negatively charged, which facilitates electrostatic interactions with chemokines that tend to have a positive charge 51. Interactions between chemokines and GAGs allow the formation of concentration gradients in solid phases 52, and the formation of solid gradients in the endothelium is a requirement to drive cell migration in response to chemokines such as CCL5 and CCL2 53. Additionally, it has been described that binding to GAGs increases the affinity of chemokines to their receptors 51; and that the dimerization or oligomerization of chemokines modifies their binding with the GAGs, by modifying the exposure of amino acid sequences with positive charges 50. *In vitro* CX3CL1 can bind to heparin 14, but there is no evidence regarding whether this interaction occurs *in vivo*, and its possible implications.

**Figure 3:**
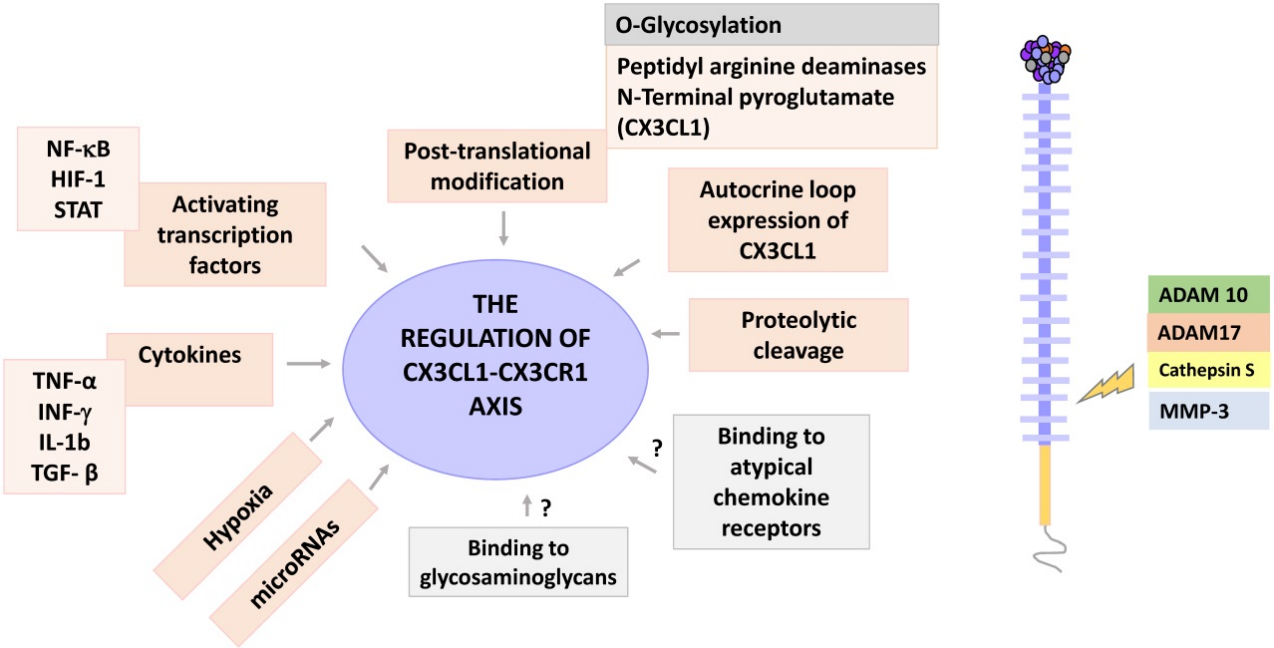
** Regulation of the CX3CL1-CX3CR1 axis.** The CX3CL1-CX3CR1 axis has several levels of regulation, including cytokine stimulation, hypoxia, activation of transcription factors, posttranscriptional modifications and proteolytic shedding. CX3CL1 is a target for the enzymes ADAM 10 and 17, Cathepsin S and some metalloproteinases, such as MMP-3. Other regulatory points for this axis may be the binding of CX3CL1 to glycosaminoglycans, as well as to atypical chemokine receptors. The potential regulation points are shown in gray.

Finally, chemokines can bind to atypical chemokine receptors, which would have the function of sequestering an excess of chemokines. Atypical chemokine receptors are 7TM receptors that bind chemokines but do not induce an activation signal 54. To date there is no data available on whether CX3CL1 binds to any of the 4 known atypical chemokine receptors (ACKR1, ACKR2, ACKR3 and ACKR4), or to GAGs. **Figure 3** shows the regulation points of the CX3CL1-CX3CR1 axis.

## Biological functions and pathological roles of CX3CL1-CX3CR1

The brain is the organ for which the most information about the CX3CL1-CX3CR1 axis is available. CX3CL1 is constitutively expressed by neurons while its receptor is expressed by microglia cells, a cell type of myeloid origin that resides throughout the nervous system. Microglia cells are specialized in the detection and elimination of damaged neurons through phagocytosis, and they produce cytokines and growth factors 55. During homeostasis, there is a constant paracrine communication between neurons expressing CX3CL1 and the microglia that expresses its receptor; this communication mediates the removal of damaged neurons 55. On the other hand, CX3CL1-CX3CR1 signaling participates in the development of the central nervous system, synaptic transmission and neuronal plasticity. For example, it has been shown that activation of this axis increases the number of synapses, neural maturation networks and neurogenesis 56; although CX3CR1 in homeostasis is basically expressed by cells of the microglia, it is also present in neurons of the hippocampus region. On adult mice, some of the new neurons have the ability to integrate with pre-existing neural networks, and it has been reported that the disruption of the CX3CL1-CX3CR1 axis decreases the survival and proliferation of neuronal progenitors in young mice 57. In neuroinflammation, the expression patterns of CX3CL1-CX3CR1 in the brain are modified: astrocytes acquire the expression of mCX3CL1, while the neuronal mCX3CL1 is shedded due to an increase in the concentration of cathepsin S. This means that during neuroinflammation, the homeostatic interaction between neurons expressing mCX3CL1 and microglia expressing the CX3CR1 receptor is lost. Microglia could then potentially be stimulated with sCX3CL1 of neuronal origin and interact with astrocytes through mCX3CL1. It has been reported that the acquisition of mCX3CL1 expression by astrocytes can also lead to the activation of microglia 58. In the murine model of stroke by occlusion of the middle cerebral artery, it was observed that 24 hours after the stroke, there was a positive regulation of CX3CR1 expression in hippocampal neurons and an increase in neuronal apoptosis, but in CX3CR1^-/-^ mice, apoptosis was diminished 59 so the disruption of the CX3CL1-CX3CR1 axis could protect against ischemic brain damage. Regarding mCX3CL1, it seems to mediate integrin-independent cellular adhesion 60.

It is known that sCX3CL1 induces survival pathways in other cell types of myeloid origin such as monocytes 61. When human monocytes are subjected to serum deprivation *in vitro*, stimulation with sCX3CL1 decreases the levels of reactive oxygen species and improves survival 61. Because only minority subpopulations of monocytes (intermediate and non-classical) express CX3CR1, the effect on their survival could be indirect, i.e. due to the pro-inflammatory cytokines that these cells produce 62. Furthermore, sCX3CL1 is chemotactic for several cell types, such as T lymphocytes, NK and DCs, and intermediate and non-classical monocytes 62, 63. CX3CL1 might be a pro-angiogenic chemokine, since it has been found that placental tissue from diabetic pregnant women, which has a higher density of microvasculature, is enriched in this chemokine 64.

On the other hand, CX3CL1 appears to be a modulator of fibrotic processes. In the model of hypertensive renal fibrosis, the expression of the CX3CL1-CX3CR1 axis was found to be increased on wild type mice, while CX3CR1^-/-^ mice presented a decrease in the expression of pro-collagen, and in infiltrating macrophages (in the presence of TGF-β); and less damage by fibrosis was seen 65. In contrast, in the model of carbon tetrachloride-induced hepatic fibrosis, it was observed that CX3CR1^-/-^ mice had an increase in the hepatic production of hydroxyproline and in the levels of alanine aminotransferase, related to increased liver damage with higher numbers of infiltrating monocytes 66. However, the final effect of CX3CL1 depends of their local concentration and the receptor density in cellular context.

Two recent studies show that myeloid CX3CR1^+^ cells could be recruited to fibrotic tissue in the lung, hence contributing to the fibrotic loop. In the first study, a murine model of bleomycin-induced pulmonary fibrosis was used. The observed upregulation of the CX3CL1-CX3CR1 axis after the bleomycin challenge was associated with intrapulmonary accumulation of pro-fibrotic M2 macrophages, while CX3CR1^-/-^ mice exhibited a reduction of M2 macrophage recruitment and collagen production 67. The second study was performed on 83 patients with interstitial lung disease (ILD), who had increased CX3CL1 levels in plasma and lung tissue. Using immunofluorescence, CX3CL1 expression was found in ciliated and bronchial epithelial cells, and accumulation of non-classical monocytes in fibroblast foci in the lung was found. Using *in vitro* migration assays it was also found that recombinant CX3CL1 drives the migration of non-classical monocytes in patients with interstitial lung disease 68.

Altogether, these results indicate that the CX3CL1-CX3CR1 axis plays a role in the elimination of damaged cells, in neurogenesis and neuroplasticity, proliferation and cell survival, and in the perpetuation of the fibrotic loop through the recruitment of an inflammatory infiltrate that promotes collagen production.

## CX3CL1-CX3CR1 in Cancer

CX3CL1-CX3CL1 overexpression has been reported on neoplastic tissue on different types of cancer, including ovarian carcinoma and gastric, pancreatic and lung cancer 69-72. Clinical and histopathological evidence on colorectal cancer (CRC) supports an anti-tumoral role of CX3CL1-CX3CR1 axis: in a recent study, the authors examined 80 histologic samples and found a positive correlation between high CX3CL1 immunoreactivity and the number of tumor infiltrating lymphocytes (TILs) including cytotoxic T cells and NK cells; a higher number of TILs is considered an indicator of better clinical prognosis 73. Similar results are observed on breast carcinoma: a high expression of CX3CL1 had a positive correlation with a higher number of stromal CD8^+^ T and NK cells, and intratumoral DCs on histological specimens. Together, these cells could inhibit tumor growth 74. Analysis of seven datasets deposited in The Cancer Genome Atlas or the Gene Expression Omnibus databases, showed that higher CX3CL1 mRNA expression is correlated with longer global survival in lung adenocarcinoma patients, but not in lung squamous cell carcinoma patients 75, suggesting that the functions of CX3CL1 could be heterogeneous even between cancer subtypes.

Studies on experimental models of carcinogenesis have evaluated the effect of CX3CR1 deficiency *in vivo*. On a murine model of melanoma using mice with a genetic deficiency in CX3CR1, the authors found larger tumors relative to their wildtype counterpart. The tumoral tissue had reduced monocyte numbers, while the number of NK cells was very similar to wildtype mice. Importantly, NK cells from the CX3CR1^- / -^ mouse exhibited altered cytokine production; for example: IFN-γ levels were decreased, while IL-6 was increased 76. In a C57BL/6 mice carcinogenesis model, where mice were inoculated with either Lewis lung carcinoma cells (3LL) or 3LL cells overexpressing CX3CL1 (3LL-FK), the authors found a decrease on tumor size in mice inoculated with 3LL-FK compared to those inoculated with 3LL WT cells. Mice inoculated with 3LL-FK had an increased infiltration of NK and DCs, and better survival 77, 78. DCs are important to antitumoral responses through the induction of effector T cells that can be recruited by CX3CL1. For example, on an experimental murine carcinogenesis model using several cancerous cell lines from different origins (colon, melanoma, LLC), the effect of DCs overexpressing CX3CL1 (DC-CX3CL1) on pre-existent tumors was examined. DC-CX3CL1 cells injected intratumorally decreased the tumor growth due to an increase of intratumoral infiltration of CD4^+^ and CD8^+^ T cells with the induction of tumor-specific CTL, and improved overall mice survival 79. On the other hand, DCs constitutively express CX3CR1 34, so it would be very interesting to evaluate in a carcinogenesis model, if immature DCs can be recruited into the tumor by exogenous CX3CL1. CX3CL1 is also able to induce migration and cytotoxicity in CX3CR1^+^ NK cells via STAT3 signaling, and *in vivo,* the blockade of the CX3CL1-CX3CR1 axis attenuated their anti-metastatic activity in hepatocellular carcinoma 38. So, antitumoral responses driven by the CX3CL1-CX3CR1 axis included the recruitment of effector cells with anti-tumoral function, and also a DC dependent activation of effector cells 77, 80.

In contrast, other studies show that the CX3CL1-CX3CR1 axis could also induce cellular functions that promote tumorigenesis and progression, such as proliferation, migration, invasion adhesion and apoptosis resistance and recruitment of immune cells with pro-tumoral activity. Regarding proliferation, on 54 surgical specimens of epithelial ovarian carcinoma a positive correlation was found on tumoral cells between immunoreactivity to CX3CL1 and a high staining intensity of Ki-67 and GILZ (glucocorticoid-induced leucine zipper), a promoter of cell cycle involved on progression and proliferation on ovarian cancer 69. On a gastric cancer cell line (DU145 cells), CX3CL1 induced cell proliferation promoting the transition to phase S of the cell cycle, and this response was inhibited with an anti CX3CL1 neutralizing antibody 81. Similar results were obtained in pancreatic cancer, supporting the role of the CX3CL1-CX3CR1 axis in promoting cell proliferation in tumoral cells 71, although this effect was not detected on a lung cancer cell line (H460 cells) 82. Furthermore, experimental evidence in cells obtained from the neu protooncogene-overexpressing transgenic mice shows that CX3CL1 promotes cell proliferation, through an indirect effect. In breast carcinoma cells from these mice, CX3CL1 induced cell proliferation by the transactivation of ErbB receptors, which in turn increased the phosphorylation of Erk, activating a pathway that has been linked to carcinogenesis 83. Another mechanism implicated in CX3CL1-induced proliferation might be the activation of anaerobic glycolysis: it has been found that anaerobic glycolysis favors proliferation by the generation of several precursors for the biosynthesis of certain biomolecules. In the pancreatic carcinoma cell lines Panc-1 and MiaPaCa-2, the activation of the CX3CL1-CX3CR1 axis increased the uptake of glucose and lactate secretion, indicating that this pathway might activate anaerobic glycolysis. Importantly, CX3CL1 stimulation induced the PI3K/Akt, MAP kinase-dependent activation of HIF-1α 84.

Sustained tumor growth requires angiogenesis, and indirect evidence indicates that CX3CL1 could play a pro-angiogenic role in cancer. In a mouse model of tumorigenesis with melanoma B16 cells, it was found that when cells were treated with RNAi against CX3CL1, tumors had a smaller size than non-treated controls where CX3CL1 expression was not modified 85. In a murine model of breast cancer, the induction of CX3CL1 seemed to increase the infiltration of CX3CR1^+^ macrophages into the tumor, as well as the vascularization of the tumor. 86. In a recent study, levels of CX3CL1 in bone marrow from patients with multiple myeloma were found to be increased, and positively correlate to a higher vascularization in the bone marrow. Furthermore, plasma from those patients stimulated angiogenesis in an *in vivo* Chick Chorioallantoic Membrane angiogenesis assay. Cases in higher clinical stages positively correlate to higher concentrations of CX3CL1 in plasma. Taken together, these results suggest that CX3CL1 could contribute to the formation and/or maintenance of an angiogenic niche able to support tumor growth through macrophage recruitment. 87. Some macrophages induce an angiogenic microenvironment through the secretion of growth factors, cytokines and chemokines with pro-angiogenic activity 88. Depletion of macrophages with clodronate liposomes or with a transgenic macrophage Fas-induced apoptosis mouse model, decrease the tumor size and metastasis number of Lewis Lung Carcinoma (LLC). In CX3CR1^-/-^ mice, injection of LLC cells led to a smaller, less vascularized primary tumor. These mice also had a diminished number of macrophages, and a lower number of metastases 89. Macrophages are generated from circulating monocytes. Non-classical monocytes exhibit angiogenic activity and are selectively accumulated in the tumor perivascular region 62. Since this subpopulation is positive for CX3CR1, this receptor could mediate their accumulation, and promote tumor growth and metastasis. Some studies indicate a synergic effect of chemokine axis on cancer. For example, macrophages co-cultured with LLC or human lung cancer cell lines up-regulated CCL2-CCR2, and CX3CL1-CX3CR1 on both macrophages and on tumoral cells 89. In addition, the CX3CL1-CX3CR1 axis induces the polarization of macrophages towards the M2 phenotype 90. Another chemokine axis that could be affected by CX3CL1 on cancer is CXCL12-CXCR4, which is involved on several aspects of carcinogenesis such as angiogenesis and organ specific metastasis 91. On chronic B-cell lymphocytic leukemia (B-CLL), CX3CR1 and soluble CX3CL1 are expressed on neoplastic cells. *In vitro* stimulation of peripheric B-CLL with CX3CL1 induced the expression of the chemokine receptor CXCR4 on an AKT-dependent form. The expression of CXCR4 allowed B-CLL chemotaxis on response to CXCL12 92.

On the other hand, *in vitro* studies indicated that CX3CL1 drives the migration of neoplastic cell 70, 93, 94. For example, exogenous CX3CL1 induces cell migration in H460, but not in H292 and A549, lung cancer cell lines with similar CX3CR1 expression levels 82. There seems to be important differences in CX3CL1 expression in several breast cancer cell lines. Using wound healing assays and transwell migration assays, CX3CL1 was found to be highly chemotactic in the MDA-MB-231 line, and migration was dependent on Src/Fak kinases. Interestingly, these cells have high levels of CX3CR1 mRNA expression 95. Studies on pancreatic cancer cells also showed that CX3CL1 is chemotactic for pancreatic ductal adenocarcinoma cells, and cell migration was decreased when cells were treated with siRNA against CX3CR1 96.

The CX3CL1-CX3CR1 axis has a direct action on molecules that facilitate invasion and metastasis, such as extracellular matrix metalloproteinases (MMPs), enzymes that have a central role on tissue remodeling. In studies carried out in the human cell line A549, it was found that knocking down CX3CL1 reduced MMP-2 and MMP-9 expression 97, and studies on pancreatic adenocarcinoma cells showed that the pharmacological inhibition of CX3CR1 with the selective antagonist JMS-17-2, decreased their motility and metastasis on a murine model of carcinogenesis 98, 99. It is important to evaluate in future research whether differential fragments of CX3CL1 are generated in the tumor microenvironment due to changes in expression patterns of metalloproteases in tissues undergoing remodeling. It is even possible that CX3CL1 could influence the remodeling of the surrounding tissue by modifying the expression patterns of remodeling enzymes.

Spinal metastases are frequent on several types of cancer. A recent study explored expression of CX3CL1 with microarrays and RT-PCR, and CX3CL1 serum levels on patients with spinal metastases compared to individuals without malignances. High levels of CX3CL1 were found particularly on lung and kidney cancer 100. That result suggests that the CX3CL1-CX3CR1 axis could be related to metastasis, but in order to prove that, it would be necessary to analyze a group with nonmetastatic cancer. The CX3CL1-CX3CR1 axis was expressed on human prostate cancer tissue, and RT-q-PCR assays indicated that expression levels are higher on spinal metastasis from prostate carcinoma than on spinal primary tumors. Functional assays using prostate carcinoma cell lines indicate that CX3CL1 induced a discreet increase of cell migration related to the activation of Src and FAK kinases 94. In addition, the authors injected PC-3 cells overexpressing CX3CR1 in immunosuppressed mice and demonstrated that *in vivo,* the CX3CL1-CX3CR1 axis is important for the development of spinal metastases 94. In gastric cancer, CX3CL1-CX3CR1 expression is increased in the tumor and in adjacent nerve cells with perineural invasion, a sign that has been associated to worse prognosis in other types of cancer. 101. CX3CR1 is also overexpressed in breast cancer spine metastasis, while CX3CL1 is expressed in spinal cancellous bone, which indicates that CX3CR1-expressing malignant cells could be attracted towards a CX3CL1 gradient to the bone 95.

**Figure 4:**
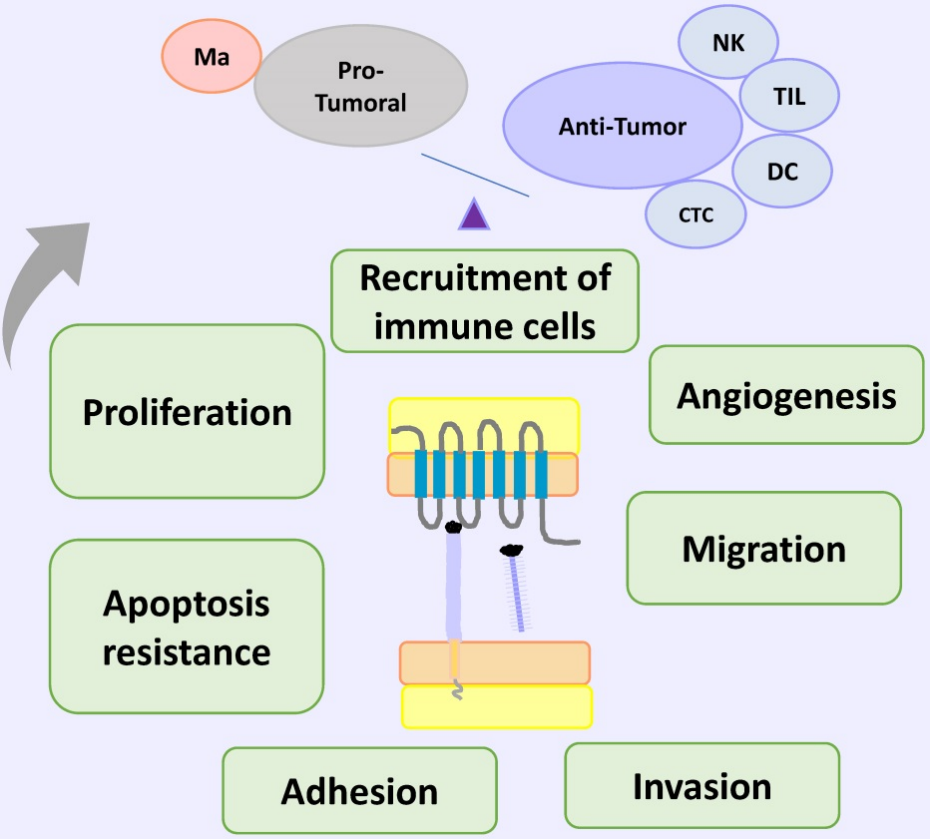
** CX3CL1-CX3CR1 on Cancer.** In cancer models, CX3CL1-CX3CR1 induced proliferation, invasion, migration, metastasis, adhesion of cells to other organs (relevant for the new niche establishment) and apoptosis resistance, that could be related to poor prognosis; and immune cell recruitment of cells with pro-tumoral and anti-tumor activities. Abbreviations: Ma, macrophages; NK, Natural killer cells; TIL, tumor-infiltrating lymphocytes; DC, dendritic cells; or cytotoxic cells.

Conditioned medium from CX3CL1-stimulated RPMI-8226 human multiple myeloma cell line induced the differentiation of osteoclasts from precursor cells, which implicates the CX3CL1-CX3CR1 axis in the progression of multiple myeloma through the generation of a niche supporting myeloma cells in the bone. On the other hand, it was also found that, after stimulation with CX3CL1, RPMI-8226 cells increased their adhesion to extracellular matrix proteins, a pathway that might be important in the development of metastases 102. However, in patients with colorectal carcinoma in clinical stages I-III, a lower expression of the CX3CL1-CX3CR1 axis in the primary tumor has been correlated to major metastasis to lymph nodes with worse prognosis; while patients with higher expression of CX3CL1-CX3CR1 in the neoplastic tissue had better 5-year survival 103. A possible interpretation is that perhaps on colorectal carcinoma, mCX3CL1 functions as an adhesion molecule that prevents metastasis. The authors found that in at least a third of the analyzed samples there is co-expression of CX3CL1-CX3CR1, in such a way that it is possible that the adhesion mediated by CX3CL1 plays an important role in the retention of neoplastic cells in the tissue of origin, and it was not associated with a greater recruitment of lymphocytes or macrophages 103. Finally, recent reports indicate that CX3CL1 induces the mesenchymal epithelial transition in prostate cancer cells, increasing their invasive and metastatic potential 104. Recent reports in human pancreatic cancer cells indicated that, on these cells, CX3CL1 is a protector against apoptosis. The authors reported than three pancreatic cancer cell lines (Aspc-1, Capan-2 and MIA PaCa-2) upregulated the expression of the anti-apoptotic molecules BCL-2 and BCL-xl in response to exogenous CX3CL1; while the pro-apoptotic caspase 3 is decreased in response to CX3CL1 stimulation. Both RNA and protein levels of Bcl-2 and Bcl-xl were decreased after siRNAs-CX3CL1 treatment. With pharmacological inhibitors, the authors demonstrated that the mechanisms are dependent on AKT phosphorylation 71. **Figure 4** and** table 1** summarize the role of CX3CL1-CX3CR1 axis in cancer.

## Concluding remarks

The chemokine axis constituted by CX3CL1-CX3CR1 is finely regulated by several mechanisms, such as specific combinations of chemokines, transcription factors, and binding to proteoglycans. Moreover, CX3CL1 has a particular level of complexity constituted by the enzymatic shedding of its chemokine domain, which is regulated by different enzymes, such as those in the ADAM family, cathepsin S, and some MMPs. In the case of metalloproteinases, signaling by the CX3CL1-CX3CR1 axis modifies the expression of this family of enzymes pointing to a putative feedback mechanism regulating the formation of sCX3CL1. In addition, O-glycosylation and binding to atypical receptors constitute points of potential regulation of the CX3CL1-CX3CR1 axis. In homeostasis, CX3CL1-CX3CR1 participates in the induction of cell survival pathways, mainly in neurons and in monocytes, where CX3CL1 functions as a remarkable anti-apoptotic molecule. On several experimental models it has been well established that this axis is important in several diseases, of which the most studied include disorders of the central nervous system and cancer, amongst others.

On cancer the CX3CL1-CX3CR1 axis has an anti-tumor role through the recruitment of anti-tumoral immune cells, such as macrophages, CD8^+^ T cells and NK cells, which can control tumor growth. In addition, accumulated evidence in pancreatic cancer, colorectal adenocarcinoma, prostate cancer and others, indicate that the CX3CL1-CX3CR1 axis also activates a pro-tumoral response related to several aspects of neoplasia, such as proliferation, migration, invasion, adhesion, apoptosis resistance and the establishment of distant metastases. The specific microenvironment, and a fine modulation of all its regulatory mechanisms, could define the global consequences of the activation of CX3CL1-CX3CR1 in a specific pathological context.

## Figures and Tables

**Table 1:**
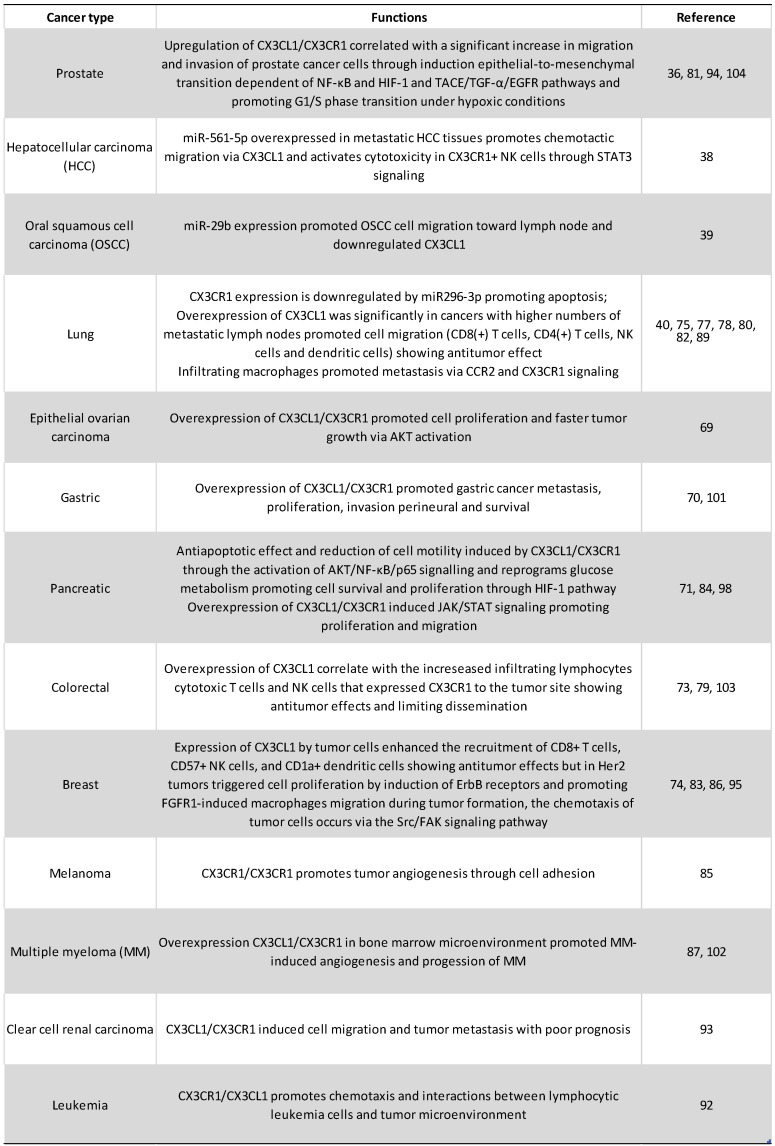
Tumorigenic functions of CX3CL1-CX3CR1 axis
